# Influenza and COVID‐19: What does co‐existence mean?

**DOI:** 10.1111/irv.12824

**Published:** 2020-10-31

**Authors:** Tawee Chotpitayasunondh, Thea Kølsen Fischer, Jean‐Michel Heraud, Aeron C. Hurt, Arnold S. Monto, Albert Osterhaus, Yuelong Shu, John S. Tam

**Affiliations:** ^1^ Queen Sirikit National Institute of Child Health Bangkok Thailand; ^2^ Nordsjaellands Hospital Hilleroed Denmark; ^3^ University of Southern Denmark Odense Denmark; ^4^ National Influenza Centre, Virology Unit Institut Pasteur de Madagascar Antananarivo Madagascar; ^5^ Virology department Institut Pasteur de Dakar Dakar Senegal; ^6^ F. Hoffmann‐La Roche Ltd. Basel Switzerland; ^7^ School of Public Health University of Michigan Ann Arbor MI USA; ^8^ Research Center for Emerging Infections and Zoonoses University of Veterinary Medicine Hannover Hannover Germany; ^9^ School of Public Health (Shenzhen) Sun Yat‐Sen University Guangzhou China; ^10^ The Hong Kong Polytechnic University Hung Hom Hong Kong

**Keywords:** antivirals, clinical management, COVID‐19, influenza, SARS‐CoV‐2, surveillance

## Abstract

The COVID‐19 pandemic caused by the novel coronavirus SARS‐CoV‐2 continues to have a major impact on healthcare and social systems throughout the world. As the clinical and epidemiological features of COVID‐19 have many parallels with influenza, it is important to ensure optimal management of both respiratory diseases as we anticipate their continued co‐circulation. In particular, there is a need to ensure that effective surveillance and diagnostic capacities are in place to monitor these and other respiratory viruses, as this will underpin decisions on the appropriate clinical management of the respective diseases. As such, we propose a series of key recommendations for stakeholders, public health authorities, primary care physicians and surveillance bodies that will help mitigate the combined risks of concurrent influenza epidemics and the COVID‐19 pandemic. We advocate the judicious use of influenza vaccines and antivirals, particularly among groups at high risk of complications, with healthcare workers also considered a priority for vaccination. It is likely that the increased use of emerging technologies such as telemedicine and contact tracing will permanently change our approach to managing infectious disease. The use of these technologies, alongside existing pharmaceutical strategies, will ensure that we achieve a holistic approach to the global public health measures needed to deal with the combined threat of influenza and COVID‐19. Ensuring that this approach is optimal will be key as we move from a reactive pandemic response towards preparing for the long‐term management of the remarkable clinical burden associated with these respiratory pathogens.

## 1 INFLUENZA IN A COVID‐19 WORLD

The recent emergence of a novel human coronavirus SARS‐CoV‐2 and the subsequent COVID‐19 pandemic is having an unrivalled impact on global healthcare and social systems. As the epidemiology, clinical manifestations and management of COVID‐19 and influenza share many features, there is a need to address and deliver targeted surveillance, diagnosis and clinical management of both respiratory diseases. SARS‐CoV‐2 and influenza viruses can realistically be expected to co‐circulate for the foreseeable future, leading to the requirement for holistic long‐term public health measures to simultaneously manage both respiratory infections and their complications effectively. The World Health Organization (WHO) has highlighted the importance of ensuring that best practices for influenza care are maintained during times of increased diversion of resources and attention to COVID‐19. It remains essential to ensure that we are well prepared to deal with future influenza seasons, with the expectation that additional COVID‐19 waves will coincide, at least in part, with circulation of seasonal influenza in the years to come. The availability of influenza vaccines and antivirals, alongside our understanding of influenza transmission dynamics, is at a state of maturity; this is in stark contrast to the situation with SARS‐CoV‐2, where currently licensed vaccines are lacking and very limited therapeutic options are available, with many features of virus transmission still unknown. As both infections result in their most severe manifestations in older adult and immunocompromised populations, the potential for co‐infection with increased severity is evident. This highlights the importance of fully optimizing available strategies for influenza management.

Here, we provide key recommendations for maintaining influenza management during the ongoing COVID‐19 pandemic. In addition, we suggest that vigilance and anticipation are required as we move from a reactive pandemic response phase towards one of preparing and planning for the likely co‐circulation of these two major infectious causes of morbidity and mortality with global impact.

## 2 TRANSMISSION DYNAMICS OF INFLUENZA VIRUSES AND SARS‐CoV‐2

To effectively prepare for ongoing co‐circulation of SARS‐CoV‐2 and influenza viruses, it is important to first appreciate the differences in transmission dynamics between the viruses. Seasonal influenza has a median basic reproduction number (R_0_) of 1.28 based on data collected over many influenza seasons^1^; this is in contrast with our early understanding of SARS‐CoV‐2, which has an estimated median R_0_ of 2.79,^2^ which has however proved to largely depend on several factors including the implementation of intervention strategies.^3^ This difference in transmission potential is likely to reflect years of prior influenza exposure and national implementation of influenza vaccination policies conferring a level of population immunity, compared with the lack of pre‐existing immunity to SARS‐CoV‐2. In countries that have experienced the first wave of COVID‐19, population estimates of exposure indicate a very large residual susceptibility. The potential for co‐circulation will vary according to social distancing measures in place to mitigate COVID‐19, population susceptibility for both viruses and vaccine availability. Importantly, this situation suggests that during seasons where influenza is freely circulating with a severe impact, COVID‐19 is likely to exert an even more pronounced effect on healthcare systems due to the overall higher transmissibility of SARS‐CoV‐2 in a susceptible population. It is vital to appreciate that holistic control measures are needed to mitigate the effects of both SARS‐CoV‐2 and influenza viruses.

## 3 EPIDEMIOLOGY, DIAGNOSIS AND SURVEILLANCE OF INFLUENZA‐LIKE ILLNESS: HOW TO EFFECTIVELY CO‐MONITOR INFLUENZA VIRUSES AND SARS‐CoV‐2

A greater understanding of the differences in epidemiology in high‐risk groups between COVID‐19 and influenza is key to ensuring optimal clinical management of each of these diseases. Children experience significant morbidity due to influenza and are considered to be key contributors to onward transmission of the virus, whereas young children infected with SARS‐CoV‐2 largely appear to be asymptomatic or have only mild symptoms.^4^, ^5^ However, our understanding of the role of children in the overall COVID‐19 disease burden and spread is still unfolding, particularly in the light of continuing uncertainties concerning their role in SARS‐CoV‐2 transmission and reports of severe immunological reactions (eg the Kawasaki‐like syndrome now termed multisystem inflammatory syndrome in Children [MIS‐C]) in infected children.^6^, ^7^ Older adults and those with underlying conditions (eg, cardiovascular, lung or kidney disease) are considered at higher risk of complications associated with COVID‐19 or influenza, and immunocompromised individuals are thought to also be at higher risk of severe disease with COVID‐19.^8^ Although pregnant women have been identified as being at high risk of complications from influenza, it is currently unclear whether these individuals are also at substantially higher risk of complications from COVID‐19.^4^, ^9^ Factors such as ethnicity, previous exposure to seasonal human coronaviruses and differences in the renin‐angiotensin‐aldosterone system (RAAS) may influence the susceptibility to SARS‐CoV‐2 infection and its complications.^10^, ^11^, ^12^

Because the majority of COVID‐19 patients present with Influenza‐Like Illness (ILI) often including fever, dry cough and fatigue, it is virtually impossible to distinguish early‐stage COVID‐19 from influenza based on symptoms alone.^4^, ^13^ This has implications for front‐line physicians who typically rely on a symptom‐based diagnosis of influenza, especially during seasonal epidemics. The co‐circulation of SARS‐CoV‐2 and influenza viruses in the next influenza season creates the need for rapid diagnostic tests for both viruses, particularly for use in high‐risk groups and at the point of presentation to healthcare professionals, as optimal treatment strategies for the two infections differ. Antiviral therapies are generally targeted at early reduction of viral replication as a means of reducing end organ damage. Both influenza and SARS‐CoV‐2 have higher viral replication at or around the time of illness onset, and any antiviral interventions will have maximum effect if delivered early in illness. Specific influenza antiviral therapy has been available for many years and should always be considered in high‐risk individuals with confirmed or suspected influenza, as per existing international guidelines^14^, ^15^, ^16^; with SARS‐CoV‐2, it will also become important to intervene early with effective antivirals, currently limited to remdesivir, to ensure optimal clinical effectiveness. We are therefore moving into an era of "test and treat" for viral respiratory infections, which requires targeted diagnosis and tailored treatment.

Surveillance data on the circulation of influenza viruses and/or SARS‐CoV‐2 and their associated diseases will help in the diagnostic and treatment decision‐making process, and public engagement in symptom reporting via mobile devices may help anticipate local or regional outbreaks. The efficacy of therapeutics for early‐stage COVID‐19 still requires further assessment, but it is likely that the majority of new antiviral therapeutics will be evaluated first in patients with more severe illness in secondary care. However, with increasing awareness of respiratory pathogens among healthcare professionals, in the future we may see a paradigm shift regarding increasing antiviral prescriptions in the primary care setting. Diagnostic testing and surveillance activities are necessary to ensure containment strategies such as isolation, contact tracing and quarantining of individuals, or containment measures in care homes or institutional/hospital settings can be implemented. Although diagnostic testing and surveillance will inevitably vary by national and regional policies, effective disease control for both viruses requires significant capacity for testing and information management, linked to implementation of control measures for COVID‐19^17^ and delivering high vaccination uptake to target populations for influenza. Innovative mechanisms to evaluate patients remotely, such as telemedicine or the use of self‐ and home monitoring, and diagnostic testing, will reduce the need for patients to leave their homes, thereby reducing the risk of further transmission.

The WHO's Global Influenza Surveillance and Response System (GISRS) has already played an important role in the response and monitoring of the spread of COVID‐19 through the establishment of sentinel surveillance networks for ILIs and severe acute respiratory infections.^18^ GISRS represents a network of public health laboratories across 125 countries with the capability to monitor virological parameters of both influenza and COVID‐19, and will likely be a crucial co‐surveillance system in the future. GISRS currently makes recommendations on influenza vaccine strain composition, which may also be applicable for COVID‐19 if the need arises. However, if COVID‐19 surveillance is to utilize existing influenza surveillance laboratories, it will be essential to ensure that capabilities, protocols and resources are revised so that ongoing influenza surveillance will be maintained while being performed side by side with SARS‐CoV‐2 surveillance. This has broad implications for low‐ and middle‐income countries, which may be struggling with COVID‐19 detection, and where resources allocated to ongoing laboratory and disease surveillance of influenza may not be sufficient to include those needed for COVID‐19. Although integration of surveillance systems for both influenza and COVID‐19 may be cost‐effective for these countries, allocating resources mainly to COVID‐19 in these settings could impact influenza surveillance. This in turn could undermine the global effort to collect and analyse influenza viruses for yearly vaccine strain selection, as well as the rapid detection of influenza viruses with pandemic potential. Recent analyses of the global incorporation of the WHO's International Health Regulations indicated suboptimal readiness to prevent, detect and respond to pandemics in ~50% of countries globally,^19^ highlighting the gaps in global health security and the importance of strengthening systems for co‐surveillance of influenza viruses and SARS‐CoV‐2 so as not to further diminish fragile health systems. Currently, the GISRS testing algorithm recommends, where resources permit, testing for both influenza virus and SARS‐CoV‐2 in parallel; if both are negative, testing for other respiratory viruses should then take place.^18^ If resources allow, an additional option would be to consider running multiplex assays for the simultaneous diagnosis of influenza, COVID‐19 and other respiratory diseases, which would be very informative particularly regarding potential co‐infections.^20^

## 4 CO‐INFECTION OF SARS‐CoV‐2 WITH INFLUENZA VIRUSES AND OTHER RESPIRATORY PATHOGENS: MUTUAL IMPACT ON CLINICAL DISEASE

Another important consideration in the clinical management of ILIs is the potential for co‐infection of SARS‐CoV‐2 with influenza virus (or other respiratory pathogens such as pneumococci), which has already been documented in up to ~30% of cases.^21^, ^22^, ^23^ The clinical impact of influenza virus and SARS‐CoV‐2 co‐infection on each corresponding disease is still largely unknown, and in the case studies reported, the use of influenza antivirals in these patients makes it difficult to assess the true impact of co‐infection without any therapeutic intervention.^23^, ^24^, ^25^ Furthermore, influenza virus circulation during the weeks preceding the outbreak in Western Europe may have "hidden" the beginning of transmission of SARS‐CoV‐2 within the community. Another factor that should be considered when influenza viruses and SARS‐CoV‐2 co‐circulate is the possible effect of "viral interference" on the prevalence and severity of the respective infections. This phenomenon has been described for the impact of rhinovirus on the circulation of pandemic influenza A/H1N1pdm09 virus in France,^26^ and at least one study has suggested that this may account for an observed "pre‐lockdown" decrease in influenza infections in Wuhan during the early COVID‐19 outbreak.^13^ Modelling work has shown that simultaneously circulating respiratory viruses including influenza, rhinoviruses and seasonal coronaviruses, can exert both positive and negative mutual effects on each other, likely affected by factors such as the innate immune response.^27^ Non‐pharmaceutical interventions (eg, social distancing, face masks, hand washing) implemented by many countries or regions to slow COVID‐19 transmission also substantially reduced influenza activity at the latter end of the 2019‐2020 northern hemisphere influenza season,^28^, ^29^, ^30^ an effect also evident during the southern hemisphere 2020 winter season.^31^ During seasonal influenza epidemics, the magnitude of these and other mitigation factors that are in place to primarily respond to COVID‐19 cases may also be expected to have an effect on influenza circulation. However, we must consider that observed decreases in influenza infections may be due to other factors such as a diversion of diagnostic resources from influenza to COVID‐19, or fewer visits to primary care sites as a result of COVID‐19 public health advice.^32^ It is imperative that physicians remain vigilant towards the threat of influenza, ahead of the winter in temperate regions but also in inter‐tropical countries with year‐round circulation, particularly if normal global travel resumes.

## 5 MAXIMIZING THE POTENTIAL OF INFLUENZA CONTROL MEASURES

The disease burden of seasonal influenza epidemics is considerable; healthcare systems tended to be stretched beyond their capacity during intensive influenza seasons.^33^ The prospect of co‐circulating influenza viruses and SARS‐CoV‐2 may lead to an even greater burden on hospital and intensive care unit (ICU) capacities and resources than has been experienced in the past months of the COVID‐19 pandemic. Therefore, adequate planning is essential to ensure sufficient resources and strategies are available to address surge capacity needs and meet the additional demand.

While it is unclear if and when effective vaccines will be available against COVID‐19, we should continue to focus on maximizing the impact of the available control measures for influenza. Influenza vaccination of risk groups and healthcare workers should be the cornerstone of seasonal influenza management. Although this is recommended by WHO and several other national and international health authorities, vaccination uptake among high‐risk groups and healthcare workers is low, and often falls far below the recommended 70% among older adults even in most high‐income countries.^34^, ^35^ Increasing influenza vaccination rates among high‐risk groups and front‐line healthcare workers is a clear and effective strategy for minimizing influenza burden and therefore allowing for better preparedness for anticipated COVID‐19 waves.^20^ Healthcare environments are prime locations for transmission of both SARS‐CoV‐2 and influenza viruses, and vaccination of healthcare workers is also key in limiting cases of nosocomial transmission. Innovative ways for promoting and delivering influenza vaccine may need to be considered if demand is greater than normal or if healthcare providers are focusing efforts on COVID‐19 cases. Furthermore, meeting the COVID‐19 associated demands of social distancing will create an additional challenge during influenza vaccination campaigns.

It is recognized that influenza vaccine effectiveness can vary greatly (eg, from 10% to 60% in the United States in recent years),^36^ whereby substantially lower effectiveness is observed in older adults or during influenza seasons when a new antigenic variant is circulating. In addition, within‐season waning of vaccine effectiveness has been documented.^37^ Therefore, there remains a key role for the use of influenza antivirals in the prophylaxis and treatment of influenza. In addition to commonly used neuraminidase inhibitors (NAIs) such as oseltamivir, the polymerase inhibitor baloxavir is an effective newer option that provides equal or improved efficacy compared to NAIs, including in patients with high risk of complications, as well as having the added convenience of a single‐dose oral regimen.^38^, ^39^ Antiviral treatment of influenza reduces disease burden and decreases both mortality and hospitalization rates,^40^, ^41^, ^42^ particularly in patients with secondary infections leading to pneumonia.^43^ Within treatment guidelines, strategies should be considered to provide high‐risk individuals with antivirals pre‐emptively, to ensure patients are treated within the 48‐hour window and healthcare systems are not overburdened. In addition to benefits associated with antiviral treatment, prophylaxis may be warranted during influenza outbreaks in care homes or institutions as an additional protective measure to reduce disease burden in the most vulnerable populations.

## 6 CONCLUDING REMARKS

The continued co‐circulation of SARS‐CoV‐2 and influenza viruses is expected to present global challenges to healthcare systems. It is important that we anticipate and consider a holistic approach to this double threat, which may present a severe challenge due to a simultaneous burden of both diseases. We propose a series of key recommendations for stakeholders, public health authorities, primary care physicians and surveillance systems that will help to mitigate the combined risks of concurrent influenza and COVID‐19 epidemics (Table 1). Maximizing the use of influenza vaccines and antivirals will lead to a reduction in influenza burden even in the absence of similar options for COVID‐19. It is likely that the COVID‐19 pandemic will lead to novel innovations in infectious disease healthcare, including rapid diagnostics and increased use of telemedicine and contact tracing, which will also mitigate the influenza burden if implemented effectively. Optimal use of these evolving technologies, combined with increased global surveillance capacities, will ensure we are better equipped to deal with future outbreaks of both influenza and COVID‐19.

**Table 1:** Key recommendations for stakeholders, public health authorities, primary care physicians and surveillance systems regarding influenza clinical management during co‐circulation of influenza viruses and SARS‐CoV‐2

Diagnosis and response
*Recommendations:* Implement combined influenza and COVID‐19 testing for all ILI patients and patients with symptoms of pneumonia Rapid testing allowing for subsequent fast contact tracing and quarantine of COVID‐19 patients as well as increasing confidence using influenza antivirals Consider early antiviral treatment for influenza patients (*see below*) Consider early antiviral treatment if diagnostic testing for influenza is not available (eg based on knowledge of levels of influenza circulating)
Surveillance
*Recommendations:* Enhance global surveillance of ILI and pneumonia with unknown aetiology Maintain influenza virus sharing and extend to SARS‐CoV‐2 to aid rapid detection of new potential pandemic strains and determination of vaccine composition Include diagnostic results for influenza and SARS‐CoV‐2 (plus other respiratory viral infections if available) in all reported ILI outbreaks Have sentinel sites participate in national, regional and international influenza virus and SARS‐CoV‐2 monitoring systems with data posted on appropriate reporting platforms Extend surveillance to co‐infections, particularly for known respiratory pathogens causing complications, such as pneumococcal *S pneumoniae or S aureus*.
Vaccination and antiviral use
*Recommendations:* Increase seasonal influenza vaccination coverage for high‐risk groups (eg older adults) and healthcare workers, as recommended by WHO and other health organizations Prepare for prophylaxis or early influenza antiviral treatment, particularly for high‐risk groups, during influenza epidemics Increase pneumococcal vaccination coverage

Abbreviations: ILI, influenza‐like illness; WHO, World Health Organization.

## CONFLICT OF INTEREST

Tawee Chotpitayasunondh, Thea Kølsen Fischer and Jean‐Michel Heraud have nothing to declare. Aeron C Hurt is employed by F. Hoffmann‐La Roche Ltd. Arnold S Monto has been a consultant and received fees from F. Hoffmann‐La Roche Ltd and Sanofi. Albert Osterhaus is CSO and co‐founder Viroclinics Biosciences and CR2O, as well as ad hoc consultant, invited speaker and SAB member to pharmaceutical companies. John S Tam has received honoraria and travel support to attend and speak at conferences by F. Hoffmann‐La Roche Ltd and Seqirus in the past 3 years. Yuelong Shu has been a consultant and received fees from Sanofi.

## AUTHOR CONTRIBUTIONS

**Tawee Chotpitayasunondh:** Writing‐original draft (equal); Writing‐review & editing (equal). **Thea Kølsen Fischer:** Writing‐original draft (equal); Writing‐review & editing (equal). **Jean‐Michel Heraud:** Writing‐original draft (equal); Writing‐review & editing (equal). **Aeron C Hurt:** Conceptualization (lead); Writing‐original draft (equal); Writing‐review & editing (equal). **Arnold Monto:** Writing‐original draft (equal); Writing‐review & editing (equal). **albert DM Osterhaus:** Writing‐original draft (equal); Writing‐review & editing (equal). **Yuelong Shu:** Writing‐original draft (equal); Writing‐review & editing (equal). **John Tam:** Writing‐original draft (equal); Writing‐review & editing (equal).

### PEER REVIEW

The peer review history for this article is available at https://publons.com/publon/10.1111/irv.12824.

## Data Availability

Data sharing is not applicable to this article as no new data were created or analysed in this study.
